# Supplementary material for: Health Risk Assessment in Children Occupationally and Para-Occupationally Exposed to Benzene Using a Reverse-Translation PBPK Model
Source: Int J Environ Res Public Health. 2023 Jan 27;20(3):2275. doi: 10.3390/ijerph20032275 (PMC9915979; doi:10.3390/ijerph20032275)
Supplement: Supplementary file 1 [file ijerph-20-02275-s001.zip › ijerph-2125369-supplementary.pdf]

**Table S1.** Parameters used to estimate the exposure to benzene in three scenarios created for shoemaker family children of Ticul, Yucatan, Mexico.

| Scenario | Parameter | Value |
|---|---|---|
| a) Children 6 to 12 years old exposed 4 hours/day | EF (days/year) | 300 |
| | ED (years) | 6 |
| | AT (days)* | 2190 |
| | ET (min/day) | 240 |
| b) Children 6 to 12 years old exposed 8 hours/day | EF (days/year) | 300 |
| | ED (years) | 6 |
| | AT (days)* | 2190 |
| | ET (min/day) | 480 |
| c) Projection for exposure during 70 years of life. | EF (days/year) | 300 |
| | ED (years) | 70 |
| | AT (days)* | 25550 |
| | ET (min/day) | 480 |

EF = exposure frequency, ED = exposure duration, AT = average exposure time, ET = exposure time
* Calculated by multiplying ED x 365 days.
